# Birth Outcomes and Natural Gas Development: McKenzie et al. Respond

**DOI:** 10.1289/ehp.1408647R

**Published:** 2014-09-01

**Authors:** Lisa M. McKenzie, Ruixin Guo, Roxana Z. Witter, David A. Savitz, Lee S. Newman, John L. Adgate

**Affiliations:** 1 Department of Environmental and Occupational Health, and; 2 Department of Biostatistics and Informatics, Colorado School of Public Health, Aurora, Colorado, USA; 3 Department of Epidemiology, Brown University, Providence, Rhode Island, USA

The comments of Fedak et al. emphasize points we made in our paper (McKenzie et al. 2014) about the importance of conducting comprehensive and rigorous research on the health effects of oil and gas development. We dedicated much of our “Discussion” to describing the limitations of our study. However, Fedak et al. have overstated these limitations.

As we stated in our paper (McKenzie et al. 2014), our study was limited by the lack of temporal and spatial specificity in using the density of existing gas wells around the maternal residence in the year of birth as the exposure. That being said, based on studies of maternal residential relocation during pregnancy, it is unlikely that a substantial proportion of subjects relocated during their pregnancy (Lupo et al. 2010; Miller et al. 2010). In addition, lack of temporal and spatial specificity of the exposure assessment would most likely have been similar for mothers with and without adverse outcomes and would have therefore resulted in weakened associations (Ritz and Wilhelm 2008; Ritz et al. 2007). Actual associations may be stronger that what we observed.

Some nondifferential exposure misclassification in the analysis of birth defects likely resulted from using data on wells existing in the birth year rather than in the year in which the first trimester of pregnancy occurred. We do not know the extent and severity of this limitation, but in many cases, it is unlikely that the density of existing wells around the maternal residence would have changed dramatically over a few months.

Our results support Fedak et al.’s statement that the most relevant benzene exposures will occur from nearby sources. Emissions from oil and gas wells are associated with the accumulation of benzene and other volatile organic compounds in the atmospheric surface layer in the general vicinity of oil and gas wells (Helmig et al. 2014). On average, one would expect more benzene emissions, and thus greater potential for benzene exposure, in areas with greater densities of natural gas wells. The results of our main analysis and sensitivity analyses indicate a linear dose response between increasing well density and the prevalence of congenital heart defects: The prevalence of congenital heart defects increases as the potential for benzene exposure increases.

Fedak et al. misinterpret our sensitivity analysis and incorrectly state that the results are insignificant. In the sensitivity analysis, we did not restrict our analysis to 1- and 5-mile radii. Rather, we restricted our exposed group to 2- and 5-mile radii. Restricting the exposure definitions would have provided stronger and more accurate associations if exposure in the narrower radii were more accurate than in the 10-mile radius. Because the restriction to the narrower radii did not markedly change the results, we can infer that the 2-, 5-, and 10-mile radii were similarly accurate.

Fedek et al. also take issue with benzene exposure as a plausible explanation for our findings because they assert that benzene is not a proven teratogen. Lack of direct evidence of causation between benzene and birth defects does not exclude the plausibility of benzene as a teratogen. Some studies have suggested an association between maternal exposure and birth defects (Lupo et al. 2011; Wennborg et al. 2005). Benzene is genotoxic, is known to cross the placenta, and has been associated with fetal demise [Agency for Toxic Substances and Disease Registry (ATSDR) 2007]. Although exposure to benzene is one plausible explanation for the observed associations, we stated in our paper that further research is needed to examine whether benzene is responsible for these associations and that other plausible explanations exist.

Fedek et al.’s comments do not change our findings or conclusions. The results of our study suggest a positive association between greater density and proximity of natural gas wells within a 10-mile radius of maternal residence and greater prevalence of congenital heart defects and possibly neural tube defects, but not oral clefts, preterm birth, or reduced fetal growth. These results and the current trends in production underscore the importance of conducting additional research on the potential health effects of oil and gas development.

## References

[r1] ATSDR (Agency for Toxic Substances and Disease Registry). (2007). Toxicological Profile for Benzene.. http://www.atsdr.cdc.gov/ToxProfiles/TP.asp?id=40&tid=14.

[r2] Helmig D Thompson CR Evans J Boylan P Hueber J Park JH 2014 Highly elevated atmospheric levels of volatile organic compounds in the Uintah Basin, Utah. Environ Sci Technol 48 4707 4715; . 10.1021/es405046r 24624890

[r3] Lupo PJ, Symanski E, Chan W, Mitchell LE, Waller DK, Canfield MA (2010). Differences in exposure assignment between conception and delivery: the impact of maternal mobility.. Paediatr Perinat Epidemiol.

[r4] Lupo P Symanski E Waller D Chan W Langlosi P Canfield M 2011 Maternal exposure to ambient levels of benzene and neural tube defects among offspring, Texas 1999–2004. Environ Health Perspect 119 397 402; 10.1289/ehp.1002212 20923742 PMC3060005

[r5] McKenzie LM Guo R Witter RZ Savtiz DA Newman LS Adgate JL 2014 Birth outcomes and maternal residential proximity to natural gas development in rural Colorado. Environ Health Perspect 122 412 417; 10.1289/ehp.1306722 24474681 PMC3984231

[r6] Miller A, Siffel C, Correa A (2010). Residential mobility during pregnancy: patterns and correlates.. Matern Child Heatlh J.

[r7] Ritz B, Wilhelm M (2008). Ambient air pollution and adverse birth outcomes: methodologic issues in an emerging field.. Basic Clin Pharmacol Toxicol.

[r8] Ritz B, Wilhelm M, Hoggatt KJ, Ghosh JKC (2007). Ambient air pollution and preterm birth in the Environment and Pregnancy Outcomes Study at the University of California, Los Angeles.. Am J Epidemiol.

[r9] Wennborg H, Magnusson L, Bonde J, Olsen J (2005). Congenital malformations related to maternal exposure to specific agents in biomedical research laboratories.. J Occup Environ Med.

